# ASBrS Keynote Address: Reimagining Stage 1 Breast Cancer—Evolution of Breast Cancer Management over the Last 120 Years

**DOI:** 10.1245/s10434-025-17849-x

**Published:** 2025-08-07

**Authors:** Gregory Bruce Mann

**Affiliations:** 1 The Royal Melbourne Hospital, Breast Service, Parkville, VIC Australia; 2 Department of Surgery, University of Melbourne, Parkville, VIC Australia

## Reimagining Stage 1 Breast Cancer

### Evolution of Breast Cancer Management over the Last 120 Years

In the late 19th century, the outlook for a patient with breast cancer was grim. Series from leading European centres reported a local recurrence rate after surgical excision of 50–70%. Patients presented with advanced disease, and arguably the best treatment was supportive care.

Dr. William Halsted published a series of patients treated with radical mastectomy in 1907, showing a local recurrence (LR) rate of 9% and additional regional recurrence in another 16%. This set the standard for treatment for the next 50 years. He presented a hypothesis that “…breast cancer … a disease that arose in one location (the breast) and, if left untreated, spread through the lymphatic system first to nearby lymph nodes and subsequently to other organs in the body”,^1^ which became known as the “Halsted hypothesis”.

Dr. Bernard Fisher developed an alternative hypothesis to that of Halsted in the 1970s, based on clinical and laboratory work showing that breast cancer did not behave as predicted by the Halsted hypothesis, and that systemic spread frequently occurred earlier in the natural history of breast cancer. Dr. Fisher hypothesized that breast cancer was a systemic disease from its inception and that tumor dissemination was not dictated by anatomic considerations but by a host-tumor relationship that determined its virulence and metastatic propensity… nuances in local-regional therapy are unlikely to have a substantial influence on ultimate survival.^2–4^

Dr. Fisher’s insights had a profound influence on breast cancer research and management. Attention was directed away from surgery to the development of effective treatment of systemic disease. The contention that breast cancer should be considered a systemic disease at the time of clinical presentation became known as the “Fisher hypothesis”.

Fisher’s alternative hypothesis led to trials of less extensive surgery, with the NSABP-B04 trial showing no survival benefit from radical mastectomy over total mastectomy and the NSABP-B06 trial showing equivalent outcomes from total mastectomy versus breast conserving surgery (BCS) with adjuvant radiotherapy (RT). BCS without RT was associated with a high locoregional recurrence rate which eventually translated into a survival disadvantage. Further trials confirmed that axillary dissection could be replaced by sentinel node biopsy (SNB), and recent trials suggest that SNB may be safely omitted in selected patients, so long as standard adjuvant therapies are delivered.

Better understanding of breast cancer biology and the development of systemic therapies has been rapid. Combination chemotherapy and adjuvant endocrine therapy (ET) led to major reductions in recurrence and deaths from breast cancer. More recently, anti-HER2 therapies, CDK inhibitors and immunotherapies have improved outcomes further, with more treatment options in the pipeline.

NCCN and other guidelines now suggest that patients with invasive breast cancer should be recommended to have RT after BCS, and that almost all patients should be recommended to have some form of adjuvant systemic therapy.^5^

Routine RT and adjuvant systemic therapy based on phenotype of the index cancer question the need for SNB in clinically stage 1 ER-positive cancer. SOUND and Insema have recently been published and support selective omission of SNB, with ASCO guidelines including this option.^6–8^

### Screening

When the Fisher hypothesis was formulated, breast cancer was diagnosed on clinical grounds. Mammography was rudimentary, and there was no organised screening for pre-symptomatic disease. Pathological assessment of specimens was quite different from what it is today, with less attention to margins, and relatively cursory attention to the axillary lymph nodes.

Dr. Phillip Strax published the results of the New York Health Plan trial of screening mammography around the same time that Fisher’s alternative hypothesis was presented.^9^ This showed that those diagnosed on screening mammography had a lower breast cancer mortality than those who were not screened. Other seminal trials of population-based mammographic screening also showed reduced breast cancer specific mortality leading to the widespread introduction of breast cancer screening.^10^

Most breast cancers identified with screening are asymptomatic as opposed to cancers in the 1970s. It is nevertheless assumed that the Fisher hypothesis remains applicable, and these cancers are considered as potentially systemic disease.

### Adjuvant Radiotherapy

Early trials of BCS without RT for Stage 1 cancer reported LR rates of 18-25%, supporting the alternative hypothesis. More focussed trials of RT omission after BCS in Stage 1, ER-positive, non-high grade cancer in older patients showed a LR rate of 10% at 10 years without RT and 1–2% with it.^11,12^ This is consistent with the hypothesis that micrometastatic spread within the breast cannot be excluded and acceptance that RT should be standard in all cases of early breast cancer after BCS.

Improved understanding of breast cancer biology has stimulated a suite of trials of de-escalation of RT using commercial gene arrays or pathological patterns indicating less aggressive biology.^13^ The presumed intent is to identify cases where dissemination in the breast can be excluded, or where disseminated cells can be eradicated by adjuvant systemic therapy. Results at 5 years follow-up suggest a low rate of LR, although ongoing recurrences beyond 5 years urge caution.^14,15^ Importantly, none of the current trials require breast imaging beyond a standard mammogram.

### Local Staging of Early Breast Cancer

Pre-operative MRI in women with apparently unifocal cancer will identify additional malignant lesions, occult to mammography, in around 15% of patients.^16^ Trials of MRI have shown a variable impact on re-excision and often an increase in mastectomy rates.^17–19^ These trials have not considered any impact on adjuvant therapies or outcomes. Commentary from 2007 that widespread use of MRI in early breast cancer will lead to mastectomy for disease that could have been treated with RT summarises prevailing attitudes,^20^ with MRI discouraged in guidelines.^21^ This is in line with the Fisher hypothesis that breast cancer should be considered systemic, with additional findings on MRI being largely clinically irrelevant.

This fundamental conception of the nature of invasive breast cancer is different from that of other cancers. Management of most other stage 1 epithelial malignancies involves less extensive treatment. Skin cancer, including melanoma, is treated with adequate wide excision without adjuvant therapy, as is colorectal, gastric, prostate, cervical, uterine, esophageal and other cancers. It is puzzling that stage 1 breast cancer in women after menopause should pose a higher risk of recurrence than early cancers in organs with far denser lymphatic and vascular networks. It is also notable that management of other cancers includes highly sensitive methods to identify synchronous primary cancers prior to excision of the known cancer.

## The Potential Impact of Using Sensitive Local Staging in Early Breast Cancer

We instigated the PROSPECT trial in 2010 to test the hypothesis that undetected occult breast cancer is responsible for LR in the absence of RT in Stage 1 non-triple negative breast cancer, and that unequivocally unifocal cancer on MRI with low-risk pathology that is adequately excised will have minimal risk of recurrence without RT.^22^

PROSPECT patients were 50 years of age or over with clinical stage 1, apparently unifocal, non-triple negative breast cancer. All underwent pre-operative MRI. BIRADS 3, 4 or 5 lesions underwent biopsy, and those with nil/minimal or mild background parenchymal enhancement on MRI and unifocal pT1N0 cancer without lymphovascular invasion or extensive DCIS, excised with a 2mm radial margin were eligible for RT omission on trial. Standard adjuvant systemic therapy was mandated for participants.

**Fig. 1:**
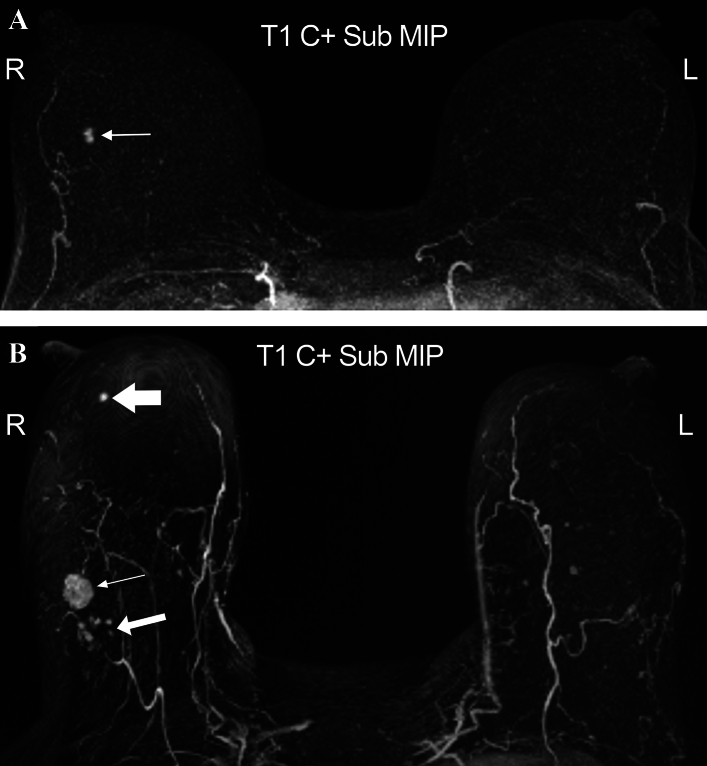
**A** 7 mm spiculated mass right breast at 11 o’clock. MRI shows minimal BPE and the unifocal index mass (arrow). Omission of RT on trial. **B** 15 mm spiculated mass right breast at 8 o’clock. MRI shows mild BPE and the index right mass (thin arrow) with adjacent occult NME (intermediate arrow). Additional ipsilateral MR-only mass at 8 o’clock (thick arrow). MR Bx of the additional ipsilateral mass = IC NST. Exclusion from omission of RT

Between September 2011 and May 2018, 443 patients with apparently unifocal cancer on standard imaging (MMG +/− US) underwent MRI. Biopsy-proven additional malignancy, separate from the index cancer, was identified and treated in 48/443 (11%) patients (Figure 1). These were ipsilateral in 7% and contralateral in 4%. Most were invasive cancer and only 11% were low grade DCIS. 63% of the ipsilateral occult cancers were > 2 cm from the edge of the index cancer. 201/443 patients had RT omitted on trial.

Primary analysis of PROSPECT, when the 100^th^ patient reached 5 years of followup, showed a LR rate without RT in the eligible group of 1% at 5 years. At primary analysis, there were only 2 LRs, one at 4.5 years and another at 7.5 years follow-up. There was a single isolated regional recurrence—presumably a false negative SLN—two contralateral new primaries and a single distance recurrence. Follow-up was available in 228/242 patients not eligible for RT omission, and there were 3 LRs and 3 contralateral primaries, but no distant recurrences in this group.

A health economic analysis showed that, in the Australian setting, the cost of additional MRIs and resultant biopsies was more than offset by savings from reduced RT use. A parallel quality of life study showed that those omitting RT on study had the expected benefits in health-related quality of life, and in addition had substantially less fear of cancer recurrence than those treated with RT, even when matched for pathology.^23,24^

Sequencing of the only case of distant metastasis and the index cancer showed these were genetically distinct, indicating it was a de novo metastatic cancer rather than a true recurrence. Thus, there were no systemic recurrences at a median of 5 years followup in this cohort of patients with lower risk cancer where highly sensitive preoperative imaging led to identification and treatment of additional disease in 11% of the cohort.^22^

## Another Alternative Hypothesis

When Fisher developed his alternative hypothesis, there was no screening. The hypothesis related to breast cancers presenting clinically. This critical fact is overlooked when the hypothesis is considered applicable to all invasive breast cancers.

The outcomes of treatment of Stage 1 breast cancer without RT in PROSPECT are consistent with an alternative hypothesis that foci of invasive cancer, occult to standard imaging, are responsible for many local and systemic recurrences occurring in those with low-risk Stage 1 breast cancer treated without RT. A certain proportion of malignant occult lesions (mOLs) would be cured by adjuvant treatment, but others would survive RT and progress during or once ET was ceased.

This is consistent with the results of the CALGB 9343 and PRIME2 trials, with many of the LRs occurring between years 5 and 10.^11,12^ In the IDEA trial, while there was only one LR in the first 5 years, there were six additional LRs reported beyond 5 years, four being in patients who were either non-adherent with ET or who had completed the recommended treatment.^15^

A subgroup of patients in the MINDACT trial with stage 1 disease were treated without ET. Their outcomes were compared to a matched group receiving ET with a total of 13.5% events in the group without and 5.2% in the group with ET. This appears to be consistent with the Fisher hypothesis, but closer examination reveals that 4.7% of the events were ipsilateral recurrences, 4.6% were contralateral primaries and 3.9% were distant events—potentially explainable in large part by occult malignancies present but not detected at initial diagnosis.^25^

The well-recognised bimodal pattern of recurrence of ER positive cancer^26^ has been attributed to the biology of these cancers and to systemic spread at the time of diagnosis. Particularly in stage 1 ER-positive breast cancer, an alternative explanation is that this is due to mOLs whose growth has been suppressed by ET.

Taken together, these data suggest that a substantial group of women with breast cancer who have been cured with BCS may be identifiable. If so, many cases of Stage 1 breast cancer can be identified where the disease is truly localised at diagnosis, where systemic therapy may reduce the risk of new primaries but will not reduce distant metastasis. This would make Stage 1 breast cancer similar to Stage 1 colorectal, gastric cancer or melanoma, where the focus after surgery is on surveillance for new primary disease rather than recurrence of the index. If such a group can be identified, women could avoid the costs and toxicities of RT, and also allow those intolerant of ET to cease it without the anxiety that they are putting their life at risk.

To achieve this goal it will be necessary to continue to confirm that patients with clinical stage 1 disease have pathological stage 1 disease. The MIRROR study of the significance of micrometastases and isolated tumour cells showed that, in the absence of systemic therapy, these are associated with a substantially poorer disease-free survival, but that this difference is not seen when the treatment is given.^27^ Implementation of the findings of SOUND and Insema may hinder exploration and implementation of de-escalation of RT and ET, committing many women to the associated toxicities to avoid the small additional morbidity of SNB.^6–8^

## Testable Consequences of Re-Imagined Stage 1 Breast Cancer

### Local Staging

If unifocal, non-TNBC is rarely systemic at diagnosis and under-treated occult malignant disease is responsible for many subsequent breast cancer events, the identification and treatment of this additional disease should allow treatment optimization and potentially improved outcomes for these patients.

We have instituted a policy of near-universal preoperative contrast-based imaging with either MRI or contrast enhanced mammography (CEM) for early breast cancer. While guidelines suggest this imaging should be used selectively and is expected to be more useful in higher stage disease, we hypothesize that identifying and treating additional disease in a patient with lower-risk index cancer is more likely to alter outcomes.

We have reported a series of 202 women with screen-detected early breast cancer having preoperative CEM showing additional DCIS or invasive cancer in 29/202 (14%), with the additional disease being considered clinically relevant in most cases.^28^ Longer term studies of a larger cohort are underway to determine whether this approach is associated with improved oncological outcomes.

### Adjuvant Radiotherapy

The PROSPECT trial findings suggest that MRI and pathology features can identify a group in whom RT can be safely omitted.^22^ The final analysis of PROSPECT will occur in May 2026 when the 100th patient treated on study without RT has reached 10 years of followup. This will be important to determine whether the substantial rate of late LRs seen in other trials is also seen in this cohort.

Confirmatory trials are essential. The PROSPECT International VErsion (PROSPECTIVE) has recently opened and will determine whether these findings can be replicated in a larger international multicentre study. Importantly, ET will not be mandatory in lower-risk patients on PROSPECTIVE, and may provide an indication whether surgery alone is appropriate in this select group of patients.

### Adjuvant Systemic Therapy

Adjuvant systemic therapy has substantially reduced mortality in Stage 2 and 3 breast cancer. While recurrence is uncommon with lower-risk phenotypes of stage 1 breast cancer, the similar relative reduction in recurrence regardless of absolute risk, coupled with the understanding that breast cancer is always potentially systemic leads to near universal recommendation for adjuvant systemic therapy. This approach is strikingly different from most other epithelial malignancies, where Stage 1 cancer is considered ‘cured’ after adequate local therapy with no role for systemic therapies.

Surveys show that adjuvant systemic therapy is responsible for significant longer term morbidity,^29^ and is the treatment that patients would most like to omit if reasonable.^30^ If occult malignant disease is responsible for many of the systemic recurrences in low-risk breast cancer, then omission of ET may be safe in those in without such disease. Exploring this possibility should be a research priority.

A combination of sensitive imaging to identify or exclude additional disease, next generation biomarkers to assess the risk of both LR and systemic recurrence and potentially circulating factors to identify groups who would benefit from systemic therapy or who can safely omit it may be key to further progress. These may be the next steps beyond the use of molecular and histological features to determine adjuvant therapy.

### Local Recurrences/Surveillance

Long-term follow up has confirmed that the breast cancer specific and overall survival rates are at least as good with BCS and RT as with mastectomy. LRs after adequate surgery and RT are seen as a marker of the underlying biology rather than a driver of any subsequent events:^31^ accordingly early detection is not critical as the outcome depends on that biology.

Recommendations for surveillance in those with a personal history of breast cancer (PHBC) are for annual mammography.^5^ Other modalities, including ultrasound and contrast-based imaging are sometimes used. There has been little research around their use; guidelines and commentary around more sensitive contrast-based imaging often discourages its use. The recent Mammo50 trial suggests that substantial de-escalation of surveillance is reasonable in selected patients over time.^32^

This understanding of subsequent local events may change if very early breast cancer is considered truly localised. As all contralateral and many ipsilateral events are unrelated to the index cancer, surveillance of those with PHBC is analogous to high-risk screening. More sensitive surveillance may lead to earlier diagnosis, better management and improved outcomes.

We have instituted a policy of routine CEM as a sole imaging modality for those with a PHBC. On the prevalent round of CEM we reported a cancer detection rate of 26/1000 with around half being detected only due to the addition of contrast.^33^ The additional cancers had pathological features suggesting they were clinically relevant. For incident round screening of 2592 surveillance episodes, the CDR was 15.4/1000 screens, again with around half being detected only due to contrast.^34^ Strikingly, the symptomatic interval cancer rate was only 0.8/1000 screens, and only 5% of cancers presented symptomatically, compared with 3.6/1000 and 20% in a meta-analysis of series of surveillance mammography.^35^

Longer term follow-up of a larger series is underway to test whether this approach results in better cancer outcomes.

### Early Detection

More effective early detection is key to both improved outcomes and successful de-escalation of therapy. Despite this, there have been few clinical trials on screening in recent decades. Other than the UK AGE trial of mammographic screening women in their 40s^36^ and the DENSE trial of MRI screening in those with very high mammographic density^37^, few RCTs in screening have been reported and none have led to widespread change in policy. Systematic reviews continue to include trials that completed accrual in the 1980s as their primary source material, despite dramatic improvements in our understanding of breast cancer risk and our ability to identify groups at higher or lower risk, as well as improvement in screening technologies^10^. This is in stark contrast to systemic therapy where many trials have progressively improved and refined adjuvant therapy.

Benefits to individuals and health systems from tailored treatment of very early breast cancer, with a large proportion of patients being safely treated with less than standard treatment and a small group being treated with more, will only be realized if a larger proportion of women destined to develop cancer are diagnosed at a very early stage. Work on implementation of risk-adjusted screening is vital and should be a high priority for the research community.

### Implications of Reimagining Stage 1 Cancer

Fisher’s alternative hypothesis regarding operable breast cancer transformed breast cancer research and treatment. De-escalation of surgery and improved systemic therapy has dramatically reduced surgical morbidity and improved outcomes. Unintended consequences may be impeding further progress in various areas. These include:

1. Ambivalence around the potential for breast cancer screening to improve outcomes.
2. Scepticism regarding the importance of local staging at the time of diagnosis
3. Acceptance that RT after BCS should be standard in almost all cases of early breast cancer
4. Acceptance that adjuvant systemic therapy should be near-universal for breast cancer
5. Acceptance that LR after treatment reflects biology and earlier diagnosis will not alter outcome.

## Summary

Breast cancer was a clinical disease when Dr. Fisher developed the alternative hypothesis. This is largely overlooked and the hypothesis is now considered applicable to all invasive breast cancers.

The outcomes of stage 1 non-TNBC treated in the PROSPECT study without RT are consistent with another alternative hypothesis, being that foci of breast cancer, occult to conventional imaging, are responsible for the substantial rates of LR, and the small incidence of systemic recurrence observed with low-risk Stage 1 breast cancer. Incorporation of preoperative contrast-based imaging to identify or exclude additional disease may be key to further progress in the treatment of very early breast cancer.

If cases of early breast cancer that is truly localised can be reliably identified, then better early detection will be key to major improvement in treatment and outcomes.

As Dr. Norman Wolmark stated in his eulogy to Dr. Fisher—“when a hypothesis gained an eponym it ceased to be a theory and became a bias”. It is time to re-assess the Fisher hypothesis in the setting of stage 1 breast cancer.^4^
